# Photochemistry of Thymine in Protic Polar Nanomeric Droplets Using Electrostatic Embeding TD-DFT/MM

**DOI:** 10.3390/molecules26196021

**Published:** 2021-10-04

**Authors:** Miquel Huix-Rotllant

**Affiliations:** Aix-Marseille University, CNRS, ICR, 13397 Marseille, France; miquel.huixrotllant@univ-amu.fr

**Keywords:** electrostatic embedding, QM/MM, conical intersections, nucleobases, thymine

## Abstract

Thymine photochemistry is important for understanding DNA photodamage. In the gas phase, thymine undergoes a fast non-radiative decay from S ${}_{2}$ to S ${}_{1}$ . In the S ${}_{1}$ state, it gets trapped for several picoseconds until returning to the ground-state S ${}_{0}$ . Here, we explore the electrostatic effects of nanomeric droplets of methanol and water on the excited states of thymine. For this purpose, we develop and implement an electrostatic embedding TD-DFT/MM method based on a QM/MM coupling defined through electrostatic potential fitting charges. We show that both in methanol and water, the mechanism is similar to the gas phase. The solvent molecules participate in defining the branching plane of S ${}_{0}$ /S ${}_{1}$ intersection and have a negligible effect on the S ${}_{1}$ /S ${}_{2}$ intersection. Despite the wrong topology of the ground/excited state intersections, electrostatic embedding TD-DFT/MM allows for a fast exploration of the potential energy surfaces and a qualitative picture of the photophysics of thymine in solvent droplets.

## 1. Introduction

Photodamage of deoxyribonucleic acid (DNA) originating from ultraviolet (UV) sunlight radiation is one of the main sources of skin cancer [1]. UV light can induce irreparable damage to the genetic code, despite the many natural protection and correction mechanisms that exist [2]. The most basic protection against UV radiation is found at the nucleobase level. Indeed, it has been shown that isolated guanine, adenine, thymine and cytosine undergo ultrafast non-radiative decays back to the ground state [3,4,5,6,7]. Photoexcitation with UV light brings the nucleobase in a singlet excited state of $\pi \pi^{*}$ character, which relaxes to lower-energy dark states of $n\pi^{*}$ character (n is a lone-pair electron of a heteroatom in the system) and finally back to the ground state.

The nucleobases decay mechanisms depend strongly on the embedding environment [8,9,10,11,12]. Indeed, when nucleobases are stacked in a DNA polymer, internal non-radiative decay competes with several charge- and proton transfers which ultimately lead to cyclobutane dimers or 6-4 photoadducts [13,14,15,16,17,18], at the origin of DNA mutations. Electronic structure combined with quantum dynamics simulations are especially suited to determine the mechanisms of deactivation in the excited state. However, there is a lack of methodologies that can treat nucleobases in their native environment at a reasonable computational cost. Especial care has to be taken at the level of theory taken for the electronic structure since the energetic order and the critical points of the photoreaction highly depend on the amount of electron correlation included in the simulations [19]. For example, three different photochemical mechanisms have been inferred from theoretical simulations of thymine: S ${}_{1}$ trapping (fast S ${}_{2}$ and slow S ${}_{1}$ decays) [20], S ${}_{2}$ trapping (slow S ${}_{2}$ and fast S ${}_{1}$ decays) [21], and S ${}_{1}$ /S ${}_{2}$ trapping (slow S ${}_{1}$ and S ${}_{2}$ decays) [22].

Recently, the effect of the electronic structure on the photochemistry of thymine in the gas phase has been studied, showing that thymine undergoes a fast non-radiative decay from S ${}_{2}$ to S ${}_{1}$ state thanks to a barrierless access to a conical intersection followed by an S ${}_{1}$ -trapping, which appears because of a barrier to reach the conical intersection back to the ground state [19]. Here, we extend the study of Ref. [19] to determine the effect of protic solvent nanomeric droplets on the photochemistry of thymine. For this purpose, we develop and implement a new quantum mechanics/molecular mechanics (QM/MM) hybrid electrostatic embedding method combining time-dependent density-functional theory (TD-DFT) [23], and classical force fields via an electrostatic potential fitting (ESPF) interaction Hamiltonian [24]. The ESPF method has been recently implemented for the analytic energy, gradient and Hessian for DFT including charge conservation and grid derivatives [25,26,27]. This method has been shown to successfully represented the electrostatic interaction between complex electrostatic environments such as proteins or solvents [28,29]. The new implementation extends these developments to the excited state, formulating the energy and analytic gradient of TD-DFT/MM required for performing both static (critical point search) and dynamic (semi-classical quantum dynamics) photochemical studies.

We apply the TD-DFT/MM method implemented here to the photochemistry of thymine in water and methanol nanomeric droplets. Despite being only qualitatively correct, TD-DFT has been shown to successfully describe the potential energy surfaces of thymine in the aqueous solution described via continuum models [9]. TD-DFT allows for a fast exploration of potential energy surface (PES) for which excited state mechanisms can be easily extracted. Indeed, we can search for minimum energy structure and approximate conical intersections via the branching update method [30]. Conical intersections are the critical points at the origin of most ultrafast non-radiative decay mechanisms, alike to the transition states in the ground state reactivity. Optimization of conical intersections is thus fundamental for determining the static picture photochemical mechanisms.

This study is organized as follows: in Section 2 the DFT/MM and TD-DFT/MM methods are described, in Section 3 the implementation and computational details are detailed, and in Section 4 the results on the photochemistry and conical intersection topology of thymine in the gas phase and in methanol and water droplets are shown. The conclusions are detailed in Section 5.

## 2. Methodology

The basics of the ESPF QM/MM electrostatic embedding coupling with ground-state self-consistent field single-determinant methods have been published and discussed in previous literature [24,26,27]. Here we followed the notation and method described in Ref. [27], in which a major reformulation of ESPF was established. Here, a formulation is presented based on ESPF that extends the QM/MM coupling to excited states. Without loss of generality, we kept the derivations using the Tamm–Dancoff approximation of the linear response TD-DFT equations (i.e., decoupled excitation and de-excitation parts of the linear response function) [31].

### 2.1. Electrostatic Embedding Coupling in ESPF DFT/MM

In the electrostatic potential fitting (ESPF) method, the QM and MM subsystems are put in contact through a purely electrostatic interaction, that can be written as

(1) $$\Delta E=\sum_{A}^{N_{\mathrm{QM}}}q_{A}\phi_{A}\;.$$

In this equation, $\phi_{A}$ is the MM electrostatic potential on the QM center *A*,

(2) $$\phi_{A}=\sum_{i}^{N_{\mathrm{MM}}}\frac{q_{i}^{\mathrm{FF}}}{\left|R_{A}-R_{i}\right|}\;,$$

where $q_{i}^{\mathrm{FF}}$ is the MM partial charge defined from the force field, and $R_{A}$ and $R_{i}$ are the Cartesian coordinates of QM and MM atoms respectively. Hereafter, we will consider only classical force fields, that is, $q_{i}^{\mathrm{FF}}$ has a fixed value without polarization. The $q_{A}$ in Equation (1) is the partial charge on the QM atom *A*, which is defined as

(3) $$q_{A}=Z_{A}-\sum_{\mu \nu }^{N_{\mathrm{AO}}}P_{\mu \nu }Q_{A,\mu \nu }\;,$$

where $Z_{A}$ is the atomic charge of atom *A*, $N_{\mathrm{AO}}$ is the number of atomic orbitals, $P_{\mu \nu }$ is the one-electron atomic-orbital density matrix, and $Q_{A,\mu \nu }$ is the electron charge population operator on QM center *A*. This operator is obtained by a fitting procedure, in which the electrostatic integrals on a grid are fitted into operators on the QM centers. This accounts to solving the equation

(4) $$\sum_{A}^{N_{\mathrm{QM}}}\frac{Q_{A,\mu \nu }}{|r_{k}-R_{A}|}=\langle \mu |\frac{1}{\left|r-r_{k}\right|}|\nu \rangle \;.$$

Using the short-hand notation $T_{k,A}={\left|r_{k}-R_{A}\right|}^{-1}$ and $V_{k,\mu \nu }=\langle \mu |\frac{1}{\left|r-r_{k}\right|}|\nu \rangle =\int dr\frac{\chi_{\mu }^{*}\left(r\right)\chi_{\nu }\left(r\right)}{|r-r_{k}|}$ , we can formally write the electron charge operators as

(5) $$Q_{A,\mu \nu }=\sum_{k}^{N_{k}}{\left[{\left(T^{†}T\right)}^{-1}T^{†}\right]}_{A,k}V_{k,\mu \nu }=\sum_{k}^{N_{k}}T_{A,k}^{+}V_{k,\mu \nu }\;.$$

Deriving Equation (1) with respect to the density matrix, we arrive at the ESPF Hamiltonian, which is given simply by

(6) $$h_{\mu \nu }=-\sum_{A}^{N_{\mathrm{QM}}}Q_{A,\mu \nu }\phi_{A}\;.$$

Due to the finite size of the grid defined in Equation (4), the charge conservation condition,

(7) $$\sum_{A}^{N_{\mathrm{QM}}}\sum_{\mu \nu }^{N_{\mathrm{AO}}}P_{\mu \nu }Q_{A,\mu \nu }=N_{\mathrm{el}}\;,$$

is not exactly satisfied. In Ref. [27], I showed that this can be imposed at the level of operators by deriving the charge conservation condition with respect to the density matrix,

(8) $$\sum_{A}^{N_{\mathrm{QM}}}Q_{A,\mu \nu }=S_{\mu \nu }\;,$$

in which $S_{\mu \nu }=\langle \mu |\nu \rangle$ is the atomic-orbital overlap matrix. This charge conservation in operator form can be imposed in the Hamiltonian directly, leading to

(9) $$h_{\mu \nu }^{\prime }=\sum_{A}^{N_{\mathrm{QM}}}Q_{A,\mu \nu }\left(\Phi_{\mathrm{av}}-\phi_{A}\right)-\Phi_{\mathrm{av}}S_{\mu \nu }\;,$$

where we introduced the average external potential,

(10) $$\Phi_{av}=\frac{1}{N_{QM}}\sum_{A}^{N_{\mathrm{QM}}}\phi_{A}\;.$$

The Hamiltonian defined in Equation (9) can thus be added to the gas phase QM Fock operator ( $F_{0}$ ) to define an electrostatic embedding formulation. The total energy of the system then becomes

(11) $$E={\mathrm{Tr}}_{\mathrm{AO}}\left[P\left(F_{0}+h^{\prime }\right)\right]+E_{\mathrm{MM}}+\sum_{A}^{N_{\mathrm{QM}}}Z_{A}\phi_{A}\;,$$

in which $E_{\mathrm{MM}}$ is the MM energy contribution and $Z_{A}$ is the atomic number of atom *A*.

The gradient of Equation (11) has two blocks with a different expression, depending on the type of atom (QM or MM) with respect to the energy is derived,

(12) ∇E0=E0xE0x˜=TrAOPF0x+h′x−TrAOWSx+EMMx+∑ANQMZAxϕAxTrAOPh′x˜+EMMx˜+∑ANQMZAxϕAx˜,

in which we symbolized the derivatives $E^{x}=\partial E/\partial x$ , in which *x* and $\tilde{x}$ represent a Cartesian coordinate of a QM or MM atom respectively. In the QM block, we substituted the derivative of the atomic-orbital density matrix with the trace of the derivative of the overlap and the energy-weighted matrix $W=PFP^{†}$ . Since the ESPF charge operator only depends on the coordinates of the grid points and the QM atoms, the derivative of the ESPF operator with respect to MM atom perturbations is simply given by

(13) TrAOPh′x˜=∑ANQMQAxΦav−ϕAx˜−Φavx˜Nel,

in which we defined $Q_{A}={\mathrm{Tr}}_{\mathrm{AO}}\left[PQ_{A}\right]$ . Thus, the gradient becomes

(14) ∇E0=E0xE0x˜=TrAOPF0x+h′x−TrAOWSx+EMMx+∑ANQMZAϕAx∑ANQMQAΦav−ϕAx˜+EMMx˜+∑ANQMZAxϕAx˜−Φavx˜Nel.

### 2.2. Electrostatic Embedding Coupling in ESPF TDA-TDDFT/MM

In TDA-TDDFT/MM, the excited state energy of state *I* is obtained by adding the excitation energy $\omega_{I}$ to Equation (11). The excitation energy is obtained by solving the eigenvalue problem,

(15) $$AX_{I}=\omega_{I}X_{I}\;,$$

in which $A_{ai,bj}=\left({\left[F_{0}+h^{\prime }\right]}_{ab}-{\left[F_{0}+h^{\prime }\right]}_{ij}\right)\delta_{ij}\delta_{ab}+\left(bj\right|f_{Hxc}\left|ai\right)$ , in which $f_{Hxc}=\delta v_{H}/\delta \rho +\delta v_{xc}/\delta \rho$ is the Hartree-exchange-correlation kernel obtained by deriving the corresponding potentials with respect to the electronic density [32].

The energy gradient is efficiently obtained by the Lagrangian approach by Furche and Ahlrichs [33]. In this approach, TDA-TDDFT/MM excitation energy gradient for state *I* is written as

(16) ∇EI=E0x+ωIxE0x˜+ωIx˜=E0x+TrPIh0+h′x−TrWISx+TrfHxΓI+TrfxcxXIXI†+∑ANQMZAϕAxE0x˜+∑ANQMQA,IΦav−ϕAx˜+∑ANQMZAxϕAx˜−Φavx˜Nel.

In this equation, $P_{I}$ and $\Gamma_{I}$ are the one- and two-electron relaxed transition densities to state *I*, $W_{I}=P_{I}FP_{I}^{†}$ and $q_{A,I}=Z_{A}-Tr\left[P_{I}Q_{A}\right]$ (for the definition of relaxed transition densities, see Ref. [33]). The relaxed densities are obtained after solving a set of coupled-perturbed equations (the Z-vector equations), which only contain two-electron terms and thus not affected by the ESPF one-electron interaction Hamiltonian [33,34].

### 2.3. Branching Update Method

In the branching update method [30], the optimization is performed by minimizing the average energy $E=\left(E_{I}+E_{J}\right)/2+{\left(E_{J}-E_{I}\right)}^{2}$ using the gradient

(17) $$\nabla E=B\nabla E_{av}+2\left(E_{I}-E_{J}\right)\nabla E_{diff}\;,$$

in which the average gradient is given by $\nabla E_{av}=\left(\nabla E_{I}+\nabla E_{J}\right)/2$ and the gradient difference is given by $\nabla E_{diff}=\left(\nabla E_{I}-\nabla E_{2}\right)/2$ . The projector *T* projects out the branching plane vectors from the average gradient, and is defined as,

(18) $$B=1-\nabla E_{diff}^{\prime }\nabla E_{diff}^{\prime T}-\nabla E_{orth}^{\prime }\nabla E_{orth}^{\prime T}\;,$$

in which $\nabla E_{orth}$ is an vector orthogonal to $\nabla E_{diff}$ . The tilde indicates that these vectors are normalized. The ESPF QM/MM, difference gradient vector between two excited states is defined as

(19) ∇EJ−EI=ωJx−ωIxωJx˜−ωIx˜=TrPJ−PIh0+h′x−TrWJ−WISx+TrfHxΓJ−ΓI+TrfxcxXJ†XJ†−XI†XI†∑ANQMΔQA,IJΦav−ϕAx˜,

in which we defined $\Delta Q_{A,IJ}=Q_{A,J}-Q_{A,I}$ . The particular case of the excited/ground state gradient, the expression for the gradient difference vector is simply the excitation energy of excited state *I*

(20) $$\nabla \left(E_{I}-E_{0}\right)=\left[\begin{array}{c} \omega_{I}^{x}\\ \omega_{I}^{\tilde{x}} \end{array}\right]=\left[\begin{array}{c} \begin{array}{c} Tr\left[P_{I}{\left(h_{0}+h^{\prime }\right)}^{x}\right]-Tr\left[W_{I}S^{x}\right]\\ +Tr\left[f_{H}^{x}\Gamma_{I}\right]+Tr\left[f_{xc}^{x}X_{I}X_{I}^{†}\right]+\sum_{A}^{N_{\mathrm{QM}}}Z_{A}\phi_{A}^{x} \end{array}\\ \sum_{A}^{N_{\mathrm{QM}}}Q_{A,I}{\left(\Phi_{av}-\phi_{A}\right)}^{\tilde{x}} \end{array}\right]\;.$$

As for the orthogonal gradient, it is estimated by a simple update procedure,

(21) $$\nabla E_{orth,k}^{\prime }=\frac{\left(\nabla E_{orth,k-1}^{\prime }\nabla E_{diff,k}^{\prime }\right)\nabla E_{diff,k-1}^{\prime }-\left(\nabla E_{diff,k-1}^{\prime }\nabla E_{diff,k}^{\prime }\right)\nabla E_{orth,k-1}^{\prime }}{\sqrt{{\left(\nabla E_{diff,k-1}^{\prime }\nabla E_{diff,k}^{\prime }\right)}^{2}+{\left(\nabla E_{orth,k-1}^{\prime }\nabla E_{diff,k}^{\prime }\right)}^{2}}}\;,$$

in which *k* indicates the optimization step.

## 3. Computational Details

The methods described here were implemented in a development version of GAMESS-US (14 FEB 2018 R1) interfaced with a modified version of Tinker 6.3.3 [35,36]. For the computation of ESPF atomic charges, an atom-centred Lebedev grid of 50 angular points and nine radial increments on each atom. Points inside the Van der Waals radii were excluded from the grid. The one-electron ESPF Hamiltonian and the derivative were added accordingly to the one-electron Hamiltonian and derivative parts of the one-electron Hamiltonian of the Fock operator. The gradients of the ground and excited states on MM atoms were computed using the corresponding ESPF atomic charges computed respectively for the density matrix and the relaxed density matrix. The environment was either optimized using the quadratic convergence optimization method of GAMESS-US or using microiterations. All calculations were performed at the TD-BH&HLYP/6-31G* using the Tamm–Dancoff approximation for the thymine atoms [37,38,39], and Amber94 for the water or methanol atoms [40].

## 4. Results and Discussion

In this section, the photochemistry of thymine was studied in gas phase and in nanometic droplets of water and methanol. The purpose was to illustrate the potential of the methodology described in Section 2 rather than performing a full mechanistic study, that would require performing quantum dynamics. The nanomeric droplets were created using the PACKMOL package [41], and subsequently optimized to the minimum energy structure of the internal QM/MM energy. For methanol and water droplets, 100 and 300 molecules respectively were included in the simulations.

### 4.1. Photochemistry of Thymine in Gas Phase

**Figure 1:**
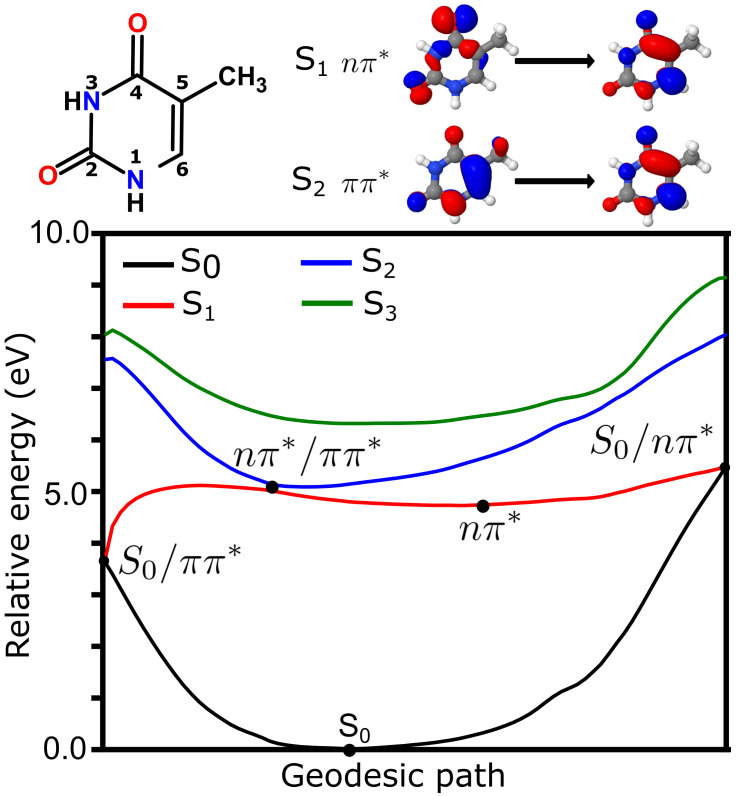
Photochemistry of thymine in the gas phase. (**top**, **left**) Schematic structure of thymine with atom numbering. (**top**, **right**) Main character of the transitions representing the first and second singlet excited states. (**bottom**) Geodesic path connecting the conical intersections between S ${}_{0}$ / $n\pi^{*}$ , S ${}_{0}$ / $\pi \pi^{*}$ and $n\pi^{*}$ / $\pi \pi^{*}$ as well as S ${}_{0}$ and $n\pi^{*}$ minima. Energies (in eV) have been computed at the TDA-TDBH&HLYP/6-31G* level.

The potential energy surface representing the key points of the photochemistry from the lowest singlet energy states of thymine in the gas phase is summarized in Figure 1. The first singlet (S ${}_{1}$ ) state was described mainly as a single excitation from a lone-pair orbital of oxygen (labeled *n*, with contributions from oxygen 4a mainly and oxygen 2a to a lesser extent) to a $\pi^{*}$ orbital delocalized mainly over carbons 4, 5 and 6. The S ${}_{2}$ state was described essentially as a π to $\pi^{*}$ transition. The photochemical pathways of deactivation of thymine have been discussed in depth in the literature [5]. Here, we focused on the topology of the potential energy surfaces (PES) and the conical intersection seams found at the TD-BH&HLYP/6-31G* level. The PES in the figure was obtained by a geodesic interpolation between the optimized structures of three conical intersections and two state minima [42]. The geodesic path represents a minimum energy path connecting the different critical points of the photoreaction, for which we can extract an idea of the photochemical mechanism. From the S ${}_{0}$ state, thymine was excited to the bright S ${}_{2}$ state of $\pi \pi^{*}$ nature. There was no stable minimum for the S ${}_{2}$ state predicted at the TD-DFT level. Rather, the S ${}_{2}$ minimum was found at the seam of conical intersection with S ${}_{1}$ , of $n\pi^{*}$ nature. From the S ${}_{1}$ /S ${}_{2}$ intersection, a branching occurs. On the left side of the graph, the path towards the intersection between the ground state and the $\pi \pi^{*}$ state is shown. This intersection could only be reached by surmounting a barrier of ca. 0.13 eV, and indeed it has been shown as a minor path of deactivation of thymine in the gas phase [5]. Most of the wavepacket would rather follow a minimum energy path towards the $n\pi^{*}$ state minimum. This minimum was long-lived, since the corresponding conical intersection was 0.8 eV higher in energy.

**Figure 2:**
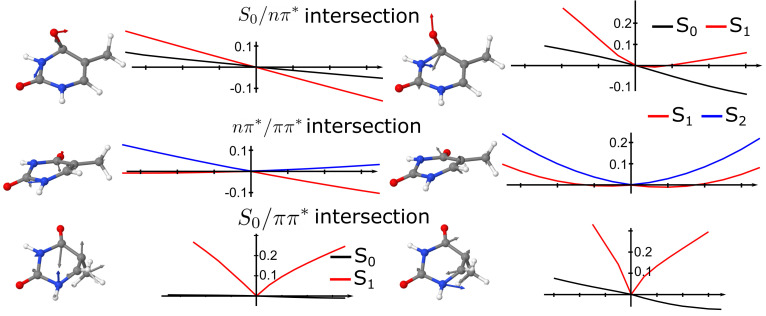
Topology of conical intersections of thymine in the gas phase. (**top**) S0/ $n\pi^{*}$ intersection, (**middle**), $n\pi^{*}$ / $\pi \pi^{*}$ intersection and (**bottom**) $n\pi^{*}$ / $\pi \pi^{*}$ intersection. Structures and graphs corresponds to the gradient difference vector and the PES along that coordinate (**left**) and non-adiabatic coupling vector (**right**).

The intersection geometries, vectors and PES around the intersection point for intersections S ${}_{0}$ / $n\pi^{*}$ , S ${}_{0}$ / $\pi \pi^{*}$ and $n\pi^{*}$ / $\pi \pi^{*}$ are represented in Figure 2. As seen from the PES along with the non-adiabatic coupling, both intersections involving the S ${}_{0}$ state were sloped conical intersections, while the $n\pi^{*}$ / $\pi \pi^{*}$ was peaked. In the S ${}_{0}$ / $n\pi^{*}$ intersection, the C ${}_{4}$ -O ${}_{4a}$ bond got perpendicular to the thymine plane, while in the S ${}_{0}$ / $n\pi^{*}$ intersection the C ${}_{5}$ -C ${}_{5}a$ bond was perpendicular to the thymine plane. The $n\pi^{*}$ / $\pi \pi^{*}$ was a boat-like structure, in which the bonds N ${}_{3}$ -H and C ${}_{6}$ -H were parallel but displaced with respect to the plane of thymine. The wrong intersection topology for the S ${}_{0}$ / $n\pi^{*}$ state was observed in the cut along the gradient difference vector, as noted previously for TDA-TD-DFT in the gas phase, in which a negative excitation energy was leading to the “true” ground state [43]. Indeed, TDA-TD-DFT led to diabatic surfaces rather than adiabatic states along the gradient difference vector.

### 4.2. Photochemistry of Thymine in Methanol Droplet

**Figure 3:**
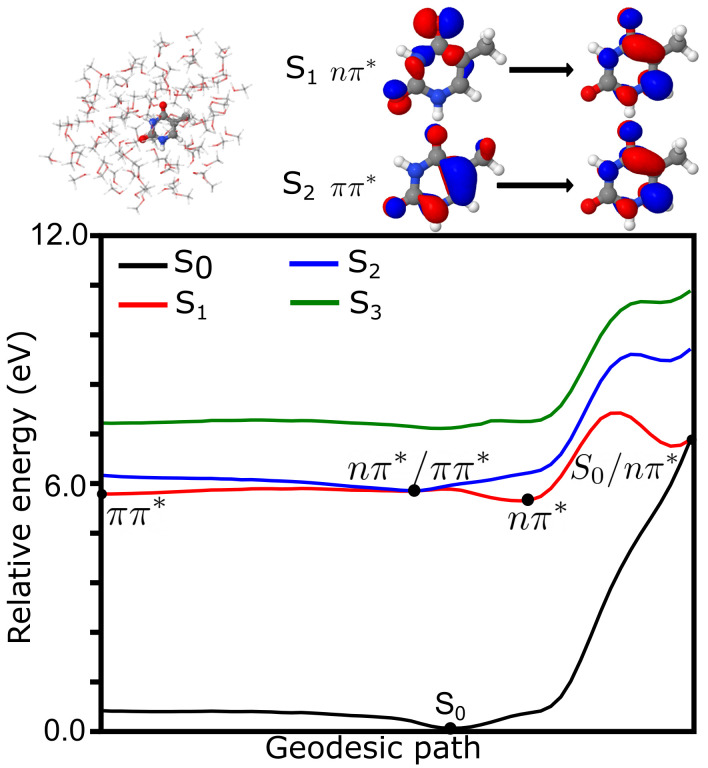
Photochemistry of thymine in methanol. (**top**, **left**) Schematic structure of thymine and methanol QM/MM model. (**top**, **right**) Main character of the transitions representing the first and second singlet excited states. (**bottom**) Geodesic path connecting the conical intersections between S ${}_{0}$ / $n\pi^{*}$ , S ${}_{0}$ / $\pi \pi^{*}$ and $n\pi^{*}$ / $\pi \pi^{*}$ as well as S ${}_{0}$ and $n\pi^{*}$ minima. Energies (in eV) have been computed at the TDA-TDBH&HLYP/6-31G* level.

The photochemistry from the lowest energy singlet states of thymine in a methanol droplet is summarized in Figure 3. The droplet contained 100 methanol molecules treated at the MM level. The state and orbital characters were the same in the gas phase. Indeed, the S ${}_{1}$ is represented by an *n* to $\pi^{*}$ transition while the S ${}_{2}$ state is represented by a π to $\pi^{*}$ transition. The PES was computed along the geodesic path connecting the $\pi \pi^{*}$ , $n\pi^{*}$ and S ${}_{0}$ minimum energy structures as well as the $n\pi^{*}$ / $\pi \pi^{*}$ and S ${}_{0}$ / $n\pi^{*}$ intersections. The initial bright state in methanol was still the S ${}_{2}$ . From this state, a barrierless access to the $n\pi^{*}$ / $\pi \pi^{*}$ intersection was observed, similar to what was observed in the gas phase. From this intersection, a branching towards the $n\pi^{*}$ and $\pi \pi^{*}$ minima occurred. From the $\pi \pi^{*}$ state, the S ${}_{0}$ / $\pi \pi^{*}$ intersection was not found. This could be since this intersection was reached by the motion of a CH ${}_{3}$ group (*vide supra*), which was hindered in solution. Rather, the motions around the $\pi \pi^{*}$ minimum could reach back to the $n\pi^{*}/\pi \pi^{*}$ minimum and thus be trapped in the $n\pi^{*}$ minimum. From the $n\pi^{*}$ minimum, a larger barrier was obtained to reach the S ${}_{0}$ / $n\pi^{*}$ than in the case of thymine in the gas phase. The difference between the $n\pi^{*}$ minimum and the corresponding intersection with the ground state was around 1.45 eV, thus the reaction was expected to be slower than in the gas phase. To reach the conical intersection, a large reorganization of the solvent molecules was needed in the path connecting these two minimum energy structures. As an estimate, the difference between the purely MM interaction energies among the solvent molecules was 0.1 eV higher in S ${}_{0}$ / $n\pi^{*}$ intersection with respect to the $n\pi^{*}$ minimum. Still, if the solvent was relaxed along this coordinate, the barrier disappeared, but the S ${}_{0}$ and the $n\pi^{*}$ states intersection disappeared. The two states had an energetic gap of around 0.4 eV with the relaxed solvent. This implies that the solvent had a direct participation in the definition of the conical intersection.

**Figure 4:**
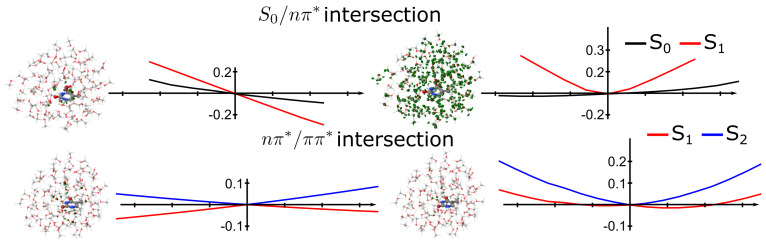
**Topology of conical intersections of thymine in methanol.** (**top**) S ${}_{0}$ / $n\pi^{*}$ intersection and (**bottom**) $n\pi^{*}$ / $\pi \pi^{*}$ intersection. Structures and graphs corresponds to the gradient difference vector and the PES along that coordinate (**left**) and non-adiabatic coupling vector (**right**). The contribution of the solvent on the branching plane vectors is shown in green.

The participation of the solvent on the intersections is clearly shown in Figure 4, in which the intersection geometries, branching plane vectors and PES around the intersection points are represented. In methanol, both the S ${}_{0}$ / $n\pi^{*}$ and $n\pi^{*}$ / $\pi \pi^{*}$ intersections peaked. On the one hand, the $n\pi^{*}$ / $\pi \pi^{*}$ intersection was completely planar, unlike the boat-like structure in the gas phase. The participation of the solvent in this intersection was negligible. On the other hand, the S ${}_{0}$ / $n\pi^{*}$ intersection was similar to the gas phase, in which the C ${}_{4}$ -O ${}_{4}a$ bond was perpendicular to the thymine bond. While the gradient vector of this intersection did not involve the solvent molecules, the approximate non-adiabatic coupling was completely delocalized over all the solvents. This implies that solvent coordinates were to be considered in the definition of the branching plane of conical intersections. Similar conclusions were extracted from model Hamiltonians of conical intersections of protonated Schiff bases in solution by Burghardt et al [44].

### 4.3. Photochemistry of Thymine in Water Droplet

**Figure 5:**
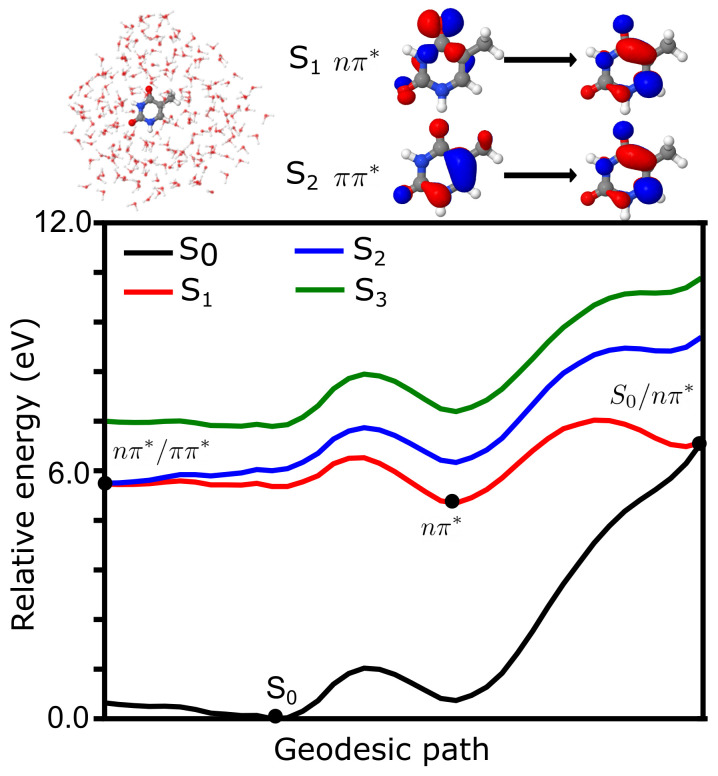
Photochemistry of thymine in water. (**top**, **left**) Schematic structure of thymine and water QM/MM model. (**top**, **right**) Main character of the transitions representing the first and second singlet excited states. (**bottom**) Geodesic path connecting the conical intersections between S ${}_{0}$ / $n\pi^{*}$ , S ${}_{0}$ / $\pi \pi^{*}$ and $n\pi^{*}$ / $\pi \pi^{*}$ as well as S ${}_{0}$ and $n\pi^{*}$ minima. Energies (in eV) have been computed at the TDA-TDBH&HLYP/6-31G* level.

The photochemistry from the lowest energy singlet states of thymine in a water droplet is summarized in Figure 5. The droplet contained 300 water molecules treated at the MM level. The state and orbital characters were the same in the gas phase and methanol droplet. The PES along the geodesic path connecting the $\pi \pi^{*}$ , $n\pi^{*}$ and S ${}_{0}$ minimum energy structures as well as the $n\pi^{*}$ / $\pi \pi^{*}$ and S ${}_{0}$ / $n\pi^{*}$ intersections is shown in the figure. From the bright S ${}_{2}$ state, a barrierless access to the $n\pi^{*}$ / $\pi \pi^{*}$ intersection was observed, similar to gas phase and methanol. At variance with methanol and similar to the gas phase, the minimum of the $\pi \pi^{*}$ state was not found in water, indicating that the minimum was close to the S ${}_{1}$ /S ${}_{2}$ intersection. Still, the S ${}_{0}$ / $\pi \pi^{*}$ intersection was not found either, probably for the same reason as in methanol, due to the hindrance of the CH ${}_{3}$ group motion due to the steric interactions with the solvent molecules. From the $n\pi^{*}$ / $\pi \pi^{*}$ intersection, all trajectories should go towards the direction of $n\pi^{*}$ minimum. A double barrier occurs in the path to the S ${}_{0}$ / $n\pi^{*}$ intersection, indicating a larger reorganization of the solvent molecules than in methanol. Indeed, from the $n\pi^{*}$ / $\pi \pi^{*}$ to the $n\pi^{*}$ minimum a first barrier was obtained, which disappeared when the solvent molecules were relaxed along the geodesic path. Additionally, from the $n\pi^{*}$ minimum a larger barrier was obtained to reach the S ${}_{0}$ / $n\pi^{*}$ than in the case of thymine in the gas phase. The difference between the $n\pi^{*}$ minimum and the corresponding intersection with the ground state was around 1.34 eV, thus the reaction was expected to be slower than in the gas phase, but slightly faster than in methanol.

**Figure 6:**
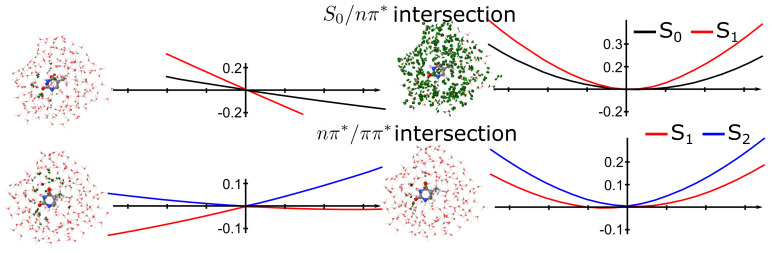
**Topology of conical intersections of thymine in water.** (**top**) S ${}_{0}$ / $n\pi^{*}$ intersection and (**bottom**) $n\pi^{*}$ / $\pi \pi^{*}$ intersection. Structures and graphs correspond to the gradient difference vector and the PES along that coordinate (**left**) and non-adiabatic coupling vector (**right**). The contribution of the solvent on the branching plane vectors is shown in green.

The participation of the solvent on the intersections is clearly shown in Figure 6, in which the intersection geometries, branching plane vectors and PES around the intersection points are represented. In water the intersections were similar to the methanol ones, both the S ${}_{0}$ / $n\pi^{*}$ and $n\pi^{*}$ / $\pi \pi^{*}$ intersections peaked. On the one hand, the $n\pi^{*}$ / $\pi \pi^{*}$ intersection was completely planar, unlike the boat-like structure in the gas phase. The participation of the solvent in this intersection was negligible. On the other hand, the S ${}_{0}$ / $n\pi^{*}$ intersection was similar to the gas phase, in which the C ${}_{4}$ -O ${}_{4}a$ bond was perpendicular to the thymine bond. While the gradient vector of this intersection did not involve the solvent molecules, the approximate non-adiabatic coupling was completely delocalized over all the solvent, as it was observed from methanol. This implies again that solvent coordinates were to be considered in the definition of the branching plane of conical intersections.

## 5. Conclusions

A new development of TD-DFT/MM energy and gradient using electrostatic potential fitting interaction Hamiltonian has been developed and implemented. This method has been used to describe the photochemistry of thymine in the gas phase and embedded in methanol and water nanomeric droplets, using critical point search in the excited states and the potential energy surface cut along a geodesic pathway connecting all minima. We show that the gas phase thymine can undergo an ultrafast relaxation pathway from the S ${}_{2}$ state to the ground state via a series of conical intersections. In solution, the barrierless path to the S ${}_{2}$ /S ${}_{1}$ intersections is similar to in the gas phase. On the contrary, the S ${}_{0}$ /S ${}_{1}$ intersections are highly dependent on the solvent state. Indeed, the approximate non-adiabatic coupling vector defining the branching plane of the S ${}_{0}$ / $n\pi^{*}$ intersection strongly mixes the motions of thymine and the solvent. The S ${}_{0}$ / $\pi \pi^{*}$ intersection was not found in solvent, but rather, a transfer from the $\pi \pi^{*}$ minimum to the $n\pi^{*}$ minimum is observed.

In conclusion, TD-DFT/MM is an efficient method to describe the excited state potential energy surface of chromophores embedded in complex media. From this study, we inferred that the $n\pi^{*}$ / $\pi \pi^{*}$ intersection is independent of the solvent state and therefore should be an equivalently fast reaction both in gas phase and in solution. On the contrary, to reach the $n\pi^{*}$ minimum and S ${}_{0}$ / $n\pi^{*}$ intersection requires a relaxation of the solvent and a particular configuration of the solvent molecules to reach the intersection. This implies that the S ${}_{0}$ / $n\pi^{*}$ intersection will be much slower in solution than in the gas phase. It remains to be seen what implication this has for the photochemistry of nucleobases in DNA. Indeed, the ground/excited intersections involve out-of-plane motions that are strongly hindered in solution, but they might still be possible in the electrostatic environment of DNA. In the future, we will use similar techniques to study the degree of delocalization of the non-adiabatic coupling vectors in DNA.

## Data Availability

All data is available upon reasonable request directly to the author.
